# Core outcomes for randomized trials and core information for clinical decision-making: implications for outcome selection

**DOI:** 10.1186/1745-6215-16-S2-P65

**Published:** 2015-11-16

**Authors:** Angus McNair, Robert Whistance, Rachel Forsythe, Rhiannon Macefield, Sara Brookes, Jane Blazeby

**Affiliations:** 1 University of Bristol, Bristol, UK; 2 University Hospitals Bristol NHS Foundation Trust, Bristol, UK

## Introduction

Core outcomes sets (COS) are an agreed minimum group of outcomes to measure in trials. Core information sets (CIS) are defined as the agreed minimum information required for clinical decision-making. Theoretically, these concepts should be closely aligned, however, there is no evidence that COSs adequately inform CISs. This study compared COS and CIS for colorectal cancer (CRC) surgery.

## Methods

All potential outcomes/information of importance were identified through systematic literature reviews, reviews of hospital information leaflets and patient interviews. This informed Delphi questionnaires which asked stakeholders (patients, surgeons and nurses) from a sample of UK CRC centres to rate the importance of 1) outcomes and 2) information on a five-point Likert scale. Respondents were resurveyed following feedback from stakeholder groups. Outcomes/information rated as less important were discarded according to pre-defined criteria. The final COS and CIS was agreed at separate international consensus meetings with professionals and patients. Comparisons were made between core set items.

## Results

Data sources identified 1216 outcome/information of CRC surgery that informed a 116 item questionnaire. Centre response rates were 79% (64/81), including 93 surgeons and 11 clinical nurse specialists, and 97 of 267 patients. Stakeholders prioritized 51 and 23 items in the first and second surveys, and consensus meetings reduced this to a 9 item COS and 10 item COS. The sets were identical apart from additional length of hospital stay information.

## Conclusion

Stakeholders largely agreed on the content of COS and CIS in CRC, but further research is needed to demonstrate this in other settings.

